# Small for Gestational Age Calves: Part I—Concept and Definition, Contributing Prenatal Factors and Neonatal Body Morphometrics in Holstein Friesian Calves

**DOI:** 10.3390/ani14142125

**Published:** 2024-07-21

**Authors:** Maya Meesters, Mieke Van Eetvelde, Karel Verdru, Jan Govaere, Geert Opsomer

**Affiliations:** Department of Internal Medicine, Reproduction and Population Medicine, Faculty of Veterinary Medicine, Ghent University, Salisburylaan 133, 9820 Merelbeke, Belgiumjan.govaere@ugent.be (J.G.); geert.opsomer@ugent.be (G.O.)

**Keywords:** prenatal programming, Holstein Friesian, small for gestational age, head sparing

## Abstract

**Simple Summary:**

Low birth weight (BW) calves experience higher mortality, lower weights at calving, reduced milk yields in their first lactation, and longer intervals before first insemination. In human medicine, small for gestational age (SGA) babies are known to suffer from increased health risks. The aim of this study was to define SGA in Holstein Friesian (HF) calves, evaluate their body measurements, and identify prenatal risk factors for being born SGA. We used models to predict calf weight based on gestation length for male and female calves from nulli- and multiparous dams. Calves with a BW below the 10th percentile were classified as SGA. We then analyzed body measurements and identified associated risk factors. SGA calves had significantly smaller body measurements and different proportions compared to average and large calves. For nulliparous dams, a higher temperature–humidity index during the 2nd trimester and older age at birth increased the birth of SGA calves. For multiparous dams, both low and high milk production during pregnancy were linked to more SGA births. This study establishes SGA in HF calves and highlights the impact of prenatal factors on calf size at birth. Further research is needed to understand the long-term effects of being born SGA on growth, reproduction and productivity.

**Abstract:**

Low birth weight (BW) calves exhibit higher mortality rates, reduced body weights at parturition, lower first-lactation milk yields, and longer parturition to first insemination intervals. In human medicine, small for gestational age (SGA) births are associated with increased perinatal morbidity and long-term metabolic risks. This study aimed to define SGA in Holstein Friesian (HF) calves, evaluate their body measurements and proportions, and identify its prenatal risk factors. Four linear regression models were built with weight as a function for gestation length for bull and heifer calves born from nulli- or multiparous dams. Calves with a BW below the 10th percentile were classified as SGA. Differences in body measurements were analyzed using ANOVA, and logistic regression models identified prenatal risk factors to be born SGA. Gestation length, calf sex, and dam parity were crucial variables in defining SGA. SGA calves had significantly smaller body measurements (*p* < 0.001) and larger body proportions (*p* < 0.001) compared to average and large calves. For nulliparous dams, a higher 2nd trimester temperature–humidity index (*p* = 0.032) and older age at parturition (>26 months, *p* = 0.026) significantly increased the birth of SGA calves. For multiparous dams, both low (<5800 kg, *p* = 0.049) and high (6700–8600 kg, *p* = 0.027) milk yields during gestation lead to more SGA births, although very high-yielding dams (>8600 kg) did not birth more SGA calves. This study establishes SGA in HF calves, suggests SGA calves are asymmetrical with evidence of “brain sparing”, and highlights the impact of prenatal factors on calf size at birth. Further research is needed to determine the long-term effects of being born SGA on growth, reproductive performance, and productivity.

## 1. Introduction

In cattle, birth size is mainly recognized as a risk factor for dystocia in terms of oversized calves [1,2] as opposed to human medicine, where there are many known risks related to the birth of small for gestational age (SGA) babies [3,4]. However, some authors have described a higher incidence of unexplained mortality in low birth weight calves [5] as well as the occurrence of more health challenges in these smaller calves in the pre-weaning period [6]. Furthermore, it has been established that birth weight is an indicator for a heifer’s predicted 400 days body weight [7] and as such may influence weight at parturition, milk yield in the first lactation, as well as the duration of the interval between parturition and first artificial insemination [8]. Depending on the applied milk feeding strategies, low birth weight calves have also been shown to remain affected by their lower weights for over two years, leading to lower 305-days milk yield and a lower fat yield during the first lactation [9]. Hence, it might be of interest to pay more attention to low birth weight calves.

Previous research described multiple factors that can influence fetal growth rate and therefore birth weight. These factors were grouped in two main categories: genetic and environmental [1]. One could classify the environmental category under the term ‘fetal programming’.

Developmental programming of fetal growth and development has been well described in both humans and animals, including cattle [10,11,12]. Developmental programming, also known as fetal programming, pertains to the notion that factors affecting growth and development during pre- and early postnatal life could lead to long-term consequences on the health and performance of the offspring [11,12].

In human medicine, intrauterine growth determines the perinatal, postnatal, and long-term health of the offspring. It has been well described that intrauterine growth restriction (IUGR) is associated with perinatal morbidity and mortality as well as with an increased risk of development of metabolic diseases in adult life [3,4,13]. Intrauterine growth restriction is commonly used interchangeably with the term SGA [3], which is used to describe a newborn with a birth weight below the 10th percentile for its gestational age [4]. IUGR can be divided into symmetrical and asymmetrical growth restriction. Symmetrical IUGR has been associated with insults during early pregnancy, where all ultrasonographic, biometric measures show abnormal growth. Conversely, asymmetrical IUGR is thought to be the result of insults occurring later in pregnancy (after the cell hyperplasia stage) with a “brain-sparing” effect [3,4,13].

In human medicine, numerous risk factors for IUGR have been identified. These have been classified in maternal, fetal, and placental factors [3,14]. Among the maternal factors, disease conditions during pregnancy, young and advanced maternal age, maternal race, smoking, and the usage of alcohol and drugs during pregnancy are well-evidenced risk factors [3]. Fetal factors can vary from genetics, infections or multiple pregnancies. Placental insufficiency, which can have multiple underlying causes, can result in suboptimal nutrition for the fetus and consequently in the birth of SGA babies [3].

In livestock, there is strong evidence that the profound effects of developmental programming can result in increased morbidity and mortality, poor growth, and reduced productivity of offspring [11]. In dairy cows, physiological conditions, such as maternal body growth, milk yield and parity, as well as environmental conditions during gestation can create a suboptimal environment for the developing fetus [12]. Both high maternal milk yield and high ambient temperatures at the end of gestation have been associated with lower birth weights [15]. Similar to what is described in humans, a lower birth weight has been associated with increased perinatal morbidity and mortality rates [16], and an increased risk of various chronic diseases throughout the offspring’s lifetime [11]. Although to the best of our knowledge, birth weight in calves has not yet been categorized as low, average or high for its gestational age.

Despite many similarities between studies on IUGR in humans and animals, the term SGA has not been defined nor has it been used in newborn calves, and to the best of our knowledge, there are only a few papers describing maternal and environmental factors influencing biometric body measurements in the early postnatal life of calves. Therefore, the first aim of the present study was to define ‘SGA’ in newborn Holstein Friesian (HF) calves, then compare body measurements and proportions of HF calves that were born with an appropriate body size with their SGA counterparts, and lastly identify the risk factors for being born SGA. We hypothesized that maternal factors such as dam age in nulliparous dams, milk yield and dry period in multiparous dams might influence the birth of SGA calves, as it has been suggested that continued growth of the dam [17], high milk yields [18] and long lactations with shorter dry period lengths [15] might negatively influence the developing fetus [12]. We also hypothesized that environmental factors such as heat stress could influence the birth of SGA calves, and that SGA calves might be asymmetrical, showing evidence of brain sparing.

## 2. Materials and Methods

All experimental procedures were approved by the Ethical Committee (EC) of the Faculty of Veterinary Medicine (Ghent University, Belgium) under the EC number 2017/87, were in accordance with the relevant guidelines and regulations and were in compliance with the ARRIVE guidelines [19].

### 2.1. Farms, Animals and Management

A first dataset from previous research performed by our group [15] was used as a training dataset. This dataset comprised data from a large dairy farm in Germany and four smaller Flemish (Belgian) farms, which was collected between August 2011 and April 2013. The German dairy farm had more than 2000 lactating cows, while the smaller Belgian farms had on average 70 lactating cows. All farms participated in official milk recording, and their average 305-days milk yield was over 9400 kg [15].

A second, smaller dataset was established by collecting data from four dairy farms in Flanders between August 2017 and November 2018. Herd size ranged from 100 to 250 lactating cows with an average 305-days milk yield of ~10,000 kg. In three herds, cows were conventionally milked twice a day, while the fourth herd used an automated milking system, recording an average of 2.6 milkings a day. All herds participated in official milk recording. Both nulliparous heifers and cows were housed in free-stall barns. In these farms, mechanical ventilation was provided for the lactating cows only.

Animals were fed according to their requirements for maintenance and production, which were based on monthly milk recordings. Rations consisted of high-quality roughages comprising maize silage, grass silage, sugar beet pulp and fodder beets supplemented with concentrates.

For reproductive management, age at first insemination in the heifers was generally around 13 to 15 months, and cows were inseminated at the first observed estrus after a 50-day waiting period. Dry period length was on average six to eight weeks, and when animals approached parturition, they were separated in a maternity pen and closely monitored by the farmer. After parturition, calves were immediately moved to individual calf pens with straw bedding and were fed two liters of colostrum. All calves were given four liters of colostrum within 12 h of birth.

### 2.2. Measurements and Data Collection

The first dataset comprised birth weight records from a total of 1594 calves born on one German and four Flemish herds. These calves were weighed within 72 h after birth using a flat scale (Seca^®^ flat scale, Seca Benelux, Naarden, The Netherlands). Inclusion criteria were that these calves were singleton, purebred HF born after a gestation length between 265 and 295 days, and that birth weight, dam parity and calf sex were recorded.

In the second dataset, a total of 615 singleton, purebred HF calves from four Flemish herds were enrolled in the study. The inclusion criteria included a gestation length between 265 and 295 days, measurement at or before ten days of age, and no missing measurements or prenatal data. Based on these inclusion criteria, 508 calves remained for further data analysis.

The following measurements were recorded for each calf from the second dataset (depicted in Supplementary File S1), and they were all performed by one person: body weight (Seca^®^ flat scale, Seca Benelux, Naarden, The Netherlands), heart girth (HG), withers height (WH), diagonal length (DL), hip width (HW), shoulder width (SW), head circumference (HC), forearm length (FA), and the length of the lower hindleg (LHL). All these measurements, except for body weight (kg), were measured in cm. HG and HC were measured with a tape measure (Animeter^®^, Albert Kerbl GmbH, Buchbach, Germany). HG was measured as the minimal circumference around the body immediately behind the elbows. HC was measured with a tape measure caudal to the supraorbital processes, at the height of the zygomatic arches, over the frontal bone and under the mandible, giving the largest circumference of the head. WH, DL, HW and SW were measured with a specially designed metal caliper (Bromet GmbH, Günzburg, Germany) on the standing animal. WH was defined as the distance from the floor to the top of the withers at the center of the shoulder, and DL was defined as the distance from the cranial edge of the *tuberculum majus humeri* to the medial border of the *tuber ischiadicum*. HW and SW were measured from hip joint to hip joint and from shoulder joint to shoulder joint, respectively. FA and LHL were measured on the recumbent calf with the same metal caliper. To measure the FA, the front leg was flexed to a 90° angle in the carpal joint, and the caliper was placed from the most cranial part of the carpal joint to the most caudal part of the elbow (*olecranon*). The LHL was measured with the fetlock flexed to a 90° angle with the caliper placed from the most cranial aspect of the fetlock to the most caudal aspect of the hock (*tuber calcaneus*).

Prenatal environment was assessed in both the broad sense of the word, meaning climatic conditions during the whole gestation, and in the narrower sense, meaning the uterine environment, thus maternal factors.

Both season of birth and parturition were divided into winter (21 December to 20 March), spring (21 March to 20 June), summer (21 June to 20 September) and fall (21 September to 20 December) as previously used by our research group [15,20,21]. To further elucidate seasonal effects, data on daily temperatures, humidity and photoperiods were recorded from the database of the Belgian Royal Meteorological Institute (RMI, Brussels, Belgium). Based on the weather data, a daily temperature–humidity index (THI) was calculated with the relative humidity (RH) and the daily maximum temperature (T), using the following formula: THI = 0.8 × T + RH × (T − 14.4) + 46.4 [22]. First, daily THI values and median weekly THI values were calculated. Then, gestation was divided into trimesters, and for each trimester, the mean THI was computed for each individual cow. Also, the amount of days above which the THI was more than 65 or more than 70 were calculated per trimester of gestation.

Dam information was extracted from the herd database and included dam parity, dam age at parturition, parturition to conception interval, calving interval, number of AIs per conception, conception date, conception season and for the nulliparous dam age at first artificial insemination (AI). Nulliparous dams were split into three age groups. The groups were defined “young”, “average” and “old” depending on their age at parturition in months, being <23 months of age, between 23 and 26 months and equal or older than 26 months, respectively. For each multiparous dam, monthly milk weights and 305-days milk yields were extracted from the herd databases, and the MilkBot^®^ model [23] was used to summarize the magnitude and shape of each lactation curve. Using this model, milk yield during the entire gestation of the dam was estimated [15,20,23]. Multiparous dams were categorized into four different production groups. Based on their milk yield during gestation, dams were divided into a “low” yielding group as well as “average”, “high” and “very high” yielding groups. These groups respectively produced <5800 kg, between 5800 and 6700 kg, between 6700 and 8600 kg or >8600 kg of milk during gestation. The dry-off date was also included to determine the dry period length. Dams with any missing or inaccuracy in AI dates or dry-off dates were excluded from the analysis.

### 2.3. Statistical Analysis

All statistical analyses were performed in R 3.6.1 [24], and all figures were generated using the ggplot2 library [25].

To define small for gestational age, a linear regression model was built, with weight as a function of gestational length. Quantile regression curves were created using the qr() function of the quantreg package to define the upper (>90%) and lower (<10%) birth weight curves and thus define each calf as small, average or large for gestational age (SGA, AGA or LGA). To assess the effects of parity and sex on being SGA, AGA or LGA, logistic regression models using the glmer() function of the ‘lme4’ package [26] were built and odds ratios were calculated. As the logistic regression model revealed major effects of dam parity and calf sex, four linear models were built to optimize the birth weight curves per subgroup: heifer and bull calf from a nulliparous dam, and heifer and bull calf from a multiparous dam. Coefficients of determination (R^2^ analogs) for the linear models were calculated using the r.squaredGLMM() function [27].

To determine the difference in body measurements and body proportions between the different gestational age groups, an ANOVA was used.

To assess the risk factors to be born SGA vs. AGA and LGA, logistic regression models using the glmer() function of the ‘lme4’ package [26] were built for nulli- and multiparous dams separately. The responsive variable was binary, with calves classified as SGA or “normal” (AGA + LGA), herd was included as a random effect in each model, and dam (dam age and milk yield group) and environmental (weekly and trimestral THI, birth season) related variables were included as fixed effects. First, univariable associations between the outcome variable and the independent risk factors were examined with statistical significance assessed at *p* < 0.15. Second, correlation coefficients were calculated between the significant variables to avoid multicollinearity in the next step. When two independent variables had a correlation coefficient > 0.60, only the one with the highest statistical significance and physiological relevance was selected for further analysis. Finally, multivariable models were built using manual forward stepwise selection in order to identify the variables that significantly improved the model. Statistical significance and tendency were declared at *p* < 0.05 and 0.05 < *p*< 0.1, respectively.

To assess the difference in age at first AI and number of AIs per conception between dam age groups (nulliparous dams) and the difference in 305-days milk yield, parturition to conception interval, and dry period length for the different milk yield groups (multiparous dams), linear models were built with herd included as a random effect.

To determine the THI threshold being significantly associated with the risk to be born SGA, a receiver operating characteristic (ROC) curve was constructed using the pROC package [28]. Based on the calculated threshold, the THI per trimester was categorized as being hot or normal. Thereafter, associations between SGA groups and the THI categories were analyzed with logistic regression models using the glmer() function of the ‘lme4’ package [26].

## 3. Results

### 3.1. Defining “Small for Gestational Age”

The first dataset included 1594 calves of which 540 (33.9%) were born out of nulliparous and 1054 (66.1%) were born from multiparous dams. Of these calves, 810 (50.8%) were female and 784 (49.2%) were male.

**Figure 1:**
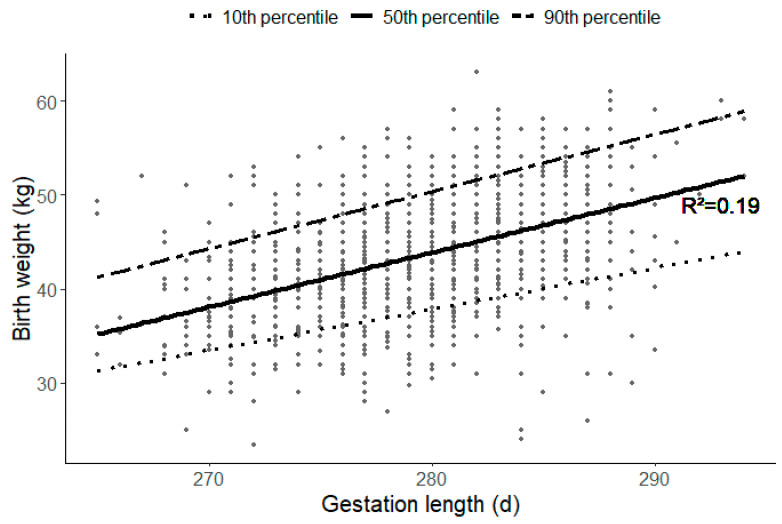
Quantile regression curves (female and male calves). The upper regression curve was defined as a 90% curve (dot–dash line) and the lower regression curve was defined as the 10% curve (dotted line). The median curve (50th percentile) is depicted as a full line.

A linear regression model was built with weight as a function of gestational length (R^2^ = 0.19). Quantile regression curves were added to predict the birth weights of each calf. The upper regression curve was defined as a 90% curve, and the lower regression curve was defined as the 10% curve (Figure 1).

Calves with a real weight above the 90% predicted weight curve were defined as large for gestational age (LGA), and calves with a real weight below 10% of the predicted weight were defined as small for gestational age (SGA). All calves with a weight between the 10th and 90th percentile were classified as average for gestational age (AGA).

The distribution of SGA, AGA and LGA calves divided for calf sex and parity of the dam is shown in Table 1. The distributions reveal a clear numerical difference within calf sex and parity categories as well as a deviating amount of SGAs and LGAs in these groups as opposed to the defined 10th and 90th percentiles. Therefore, logistic regression models were built, and odds ratios were calculated to assess the significance of the effect of both calf sex and parity on the birth of small (SGA) or normal (AGA + LGA) calves.

**Figure 2:**
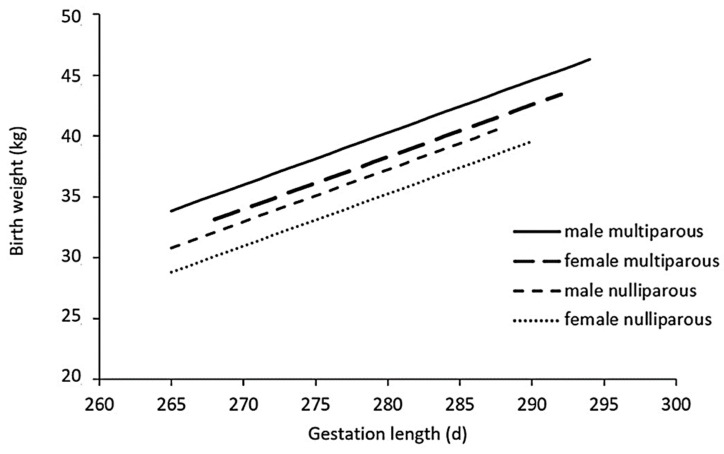
Quantile regression curves of the 10th percentile weights for each subgroup: male or female calves born out of multi- or nulliparous dams. Male calves from multiparous dams (full line) have higher 10th percentile weights than female calves from multiparous (long dash) and male (short dash) or female (dotted) calves from nulliparous dams.

Based on the logistic regression models, both calf sex and dam parity have significant effects on the birth of SGA calves. The odds ratio of being born SGA is 4.1 times higher when the dam is nulliparous (*p* < 0.001), and when the calf is born as a heifer, the OR of being SGA is 2.2 (*p* < 0.001). Thus, a new, optimized linear regression model was built based on the first, large dataset, with weight as a function of gestational age, calf sex and dam parity (R² = 0.34), and quantile regression curves of the 10th percentile weights were calculated for each subgroup (Figure 2).

The optimized model shows a more equal distribution of SGA, AGA and LGA calves within calf sex and parity categories as well as the number of SGA and LGA calves in each group corresponding to the defined 10th and 90th percentiles (Table 2).

To illustrate the difference of classifying calves according to their gestational age category instead of using birth weight only, a cross-table was made (Table 3). First, to define the 10th and 90th percentiles using only birth weight, the first, large dataset (N = 1594) was used. All calves born with a birth weight below the 10th percentile (≤36.5 kg) were classified as “low birth weight” (Low BW), and all calves with a birth weight above the 90th percentile (≥51 kg) were classified as “high birth weight” (High BW). All calves between the 10th and 90th percentiles were classified as being born with an “average birth weight” (Average BW). Secondly, the second and smaller dataset (N = 508) was used to compare the calves’ classification using previously defined birth weight groups or their gestational age group based on the optimized linear regression model.

As depicted in Table 3, 73 out of 508 (14.4%) calves were classified differently based on gestational age group vs. birth weight group. Also, 18 “Low BW” calves were actually classified as AGA and 20 “Average BW” were classified as LGA. More interestingly, one third of the calves that were classified as SGA would go unnoticed as “small” using only birth weight categories, since 30.8% of SGA calves were classified as “Average BW” using the birth weight groups.

### 3.2. Body Measurements and Proportions in SGA vs. AGA and LGA Calves

To assess the effect of gestational age on the morphometrics of newborn Holstein Friesian calves, the second dataset was used (N = 508). In this dataset, 54% (273/508) of calves were female and 46% (235/508) were male, and almost two thirds (64%, 325/508) were born out of multiparous dams.

The calves from the second dataset were defined as SGA, AGA or LGA based on the optimized model calculated in the previous section (based on the first dataset). Thus, 15% (78/508) of the calves were classified as SGA, 78% (398/508) as AGA and 6% (32/508) as LGA.

Based on an ANOVA, all body measurements were significantly smaller in SGA calves compared to their AGA counterparts. Also, LGA calves had significantly larger body measurements in comparison to the AGA calves. All body proportions were significantly different between SGA and AGA calves. However, LGA calves did not differ significantly from their AGA counterparts in most body proportions (Table 4).

### 3.3. Risk Factors to Be Born SGA

Considering most body proportions were not significantly different between AGA and LGA calves, risk factors on SGA were compared between SGA and “normal” (=AGA + LGA, N = 476) calves. Two logistic regression models were built to assess the risk factors for birth of SGA calves in either nulli-or multiparous dams.

#### 3.3.1. Nulliparous Dams

Heifers in the old group were on average 470 ± 47.3 days old at first artificial insemination (AI), which is 29.3 days older than the average group (441 ± 29.3 days, *p* = 0.002) and 90.7 days older than heifers in the young group (380 ± 21.5 days, *p* < 0.001). In addition, old heifers needed 2.6 ± 1.20 AIs for pregnancy compared to 1.7 ± 1.08 AIs in average aged (*p* = 0.001) and 1.4 ± 0.68 AIs in young heifers (*p* < 0.001). The number of AIs in the average aged dams was not significantly different from that in the young dams, although numerically, the young dams needed fewer inseminations to become pregnant.

In nulliparous dams, dam age at parturition is significantly associated with the birth of SGA calves (Table 5). Old heifers have 4.22 greater odds (*p* = 0.021) of giving birth to SGA calves compared to average aged dams. The only environmental factor significantly affecting the birth of SGA calves was a higher mean THI during the second trimester of gestation (*p* = 0.032).

**Figure 3:**
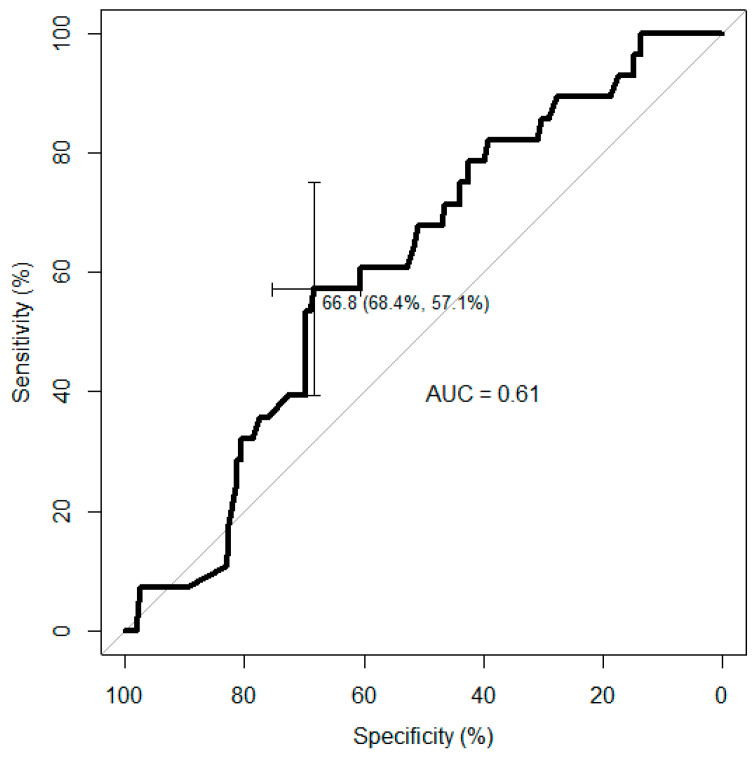
Receiver operating characteristic (ROC) curve for the second trimester temperature–humidity index (THI) and the risk of being born small for gestational age in calves from nulliparous dams, revealing a THI threshold of 66.8 (sensitivity of 55.6%, specificity of 68.8%).

Based on the ROC curve analysis, the second trimester THI threshold was set at 66.8 (Figure 3).

Based on the threshold, the second trimester THI was categorized as being hot (≥66.8) or normal (<66.8). The logistic regression model with being SGA or not as an outcome variable and the second trimester THI category (hot vs. normal) as an explanatory variable revealed that calves born after a hot (THI ≥ 66.8) second trimester of gestation have 2.75 higher odds of being born SGA (*p* = 0.0205) compared to those born after a normal (THI < 66.8) second trimester.

#### 3.3.2. Multiparous Dams

Both cows yielding low (<5800 kg) or high (6700–8600 kg) amounts of milk during gestation had significantly higher odds of giving birth to an SGA calf in comparison to cows from the average producing group (Table 6). Cows producing very high milk yields (>8600 kg) during gestation did not significantly differ from the average producing group in terms of giving birth to SGA offspring.

The 305-days milk yield significantly differed in all groups (*p* < 0.001). In the low group, 305-days milk yield was 7840 ± 1519 kg compared to 8936 ± 996 kg in the average group, 10,160 ± 908 kg in the high group, and 12,403 ± 1494 kg in the very high producing group.

Cows in the low milk yield group had a parturition to conception interval of 125 ± 89.4 days, which was significantly longer than that in the average (96 ± 41.9, *p* = 0.018), high (94 ± 56.5, *p* = 0.007), and very high (80 ± 38.7, *p* < 0.001) groups. Numerically, the shortest parturition to conception interval was observed in cows with a very high milk yield, but this was not significantly different from the average or high group.

Dams from the low milk yield group had an average dry period of 50 ± 17.8 days, which is significantly longer than that in the average (44 ± 10.7, *p* = 0.023), high (43 ± 8.4, *p* = 0.003) and very high (41 ± 7.8, *p* < 0.001) milk yield groups. Cows with a very high milk yield during gestation had the numerically shortest dry period length, although this was not significantly different from the average or high group production groups.

## 4. Discussion

Intrauterine growth determines development perinatally, postnatally and in adult life, in humans and cattle [3,11]. In humans, intrauterine growth retardation leading to low birth weight babies has been associated with an increased risk of metabolic diseases later in life [3,10]. However, while mortality due to dystocia in oversized calves is well known, mortality in low birth weight calves is often overlooked [1]. More recently, it has been shown that lower birth weight dairy calves experience more health challenges over the pre-weaning period, causing reduced average daily gains and thus lower weaning weights [6]. There are many similarities between human and animal studies on intrauterine growth retardation [12]; however, the well-known human concept of “small for gestational age” has not yet been described in cattle.

Human infants have long been classified as SGA, AGA or LGA [29]; however, to the best of our knowledge, this is the first study describing “small for gestational age” in Holstein Friesian calves.

The first objective of this study was to define “small for gestational age”, similarly to what is described in human medicine. We found that to classify Holstein Friesian calves as small, average or large for gestational age, not only birth weight and gestation length need to be included, but also calf sex and dam parity should be taken into account when creating the 10th and 90th percentile birth weight curves. As such, calves were defined as being SGA, AGA or LGA when their real birth weight was below the 10th, between the 10th and 90th or above the 90th percentile, respectively. The importance of taking gestation length into account was demonstrated when comparing calves using birth weight groups versus gestational age groups. We found that 30.8% of calves with an “average birth weight” were actually classified as SGA. The first, large training dataset consisted of 1594 calves weighed within the first 72 h after birth, whereas our second, smaller dataset (N = 508) included calves that were weighed and measured within the first 10 days after birth, which is a shortcoming in our study, as a calf weighed at 10 days may of course have gained weight since it was born. However, it has been previously described that calves kept in a conventional system (meaning no ad libitum milk or calves kept with their dam) only gain 4.5 kg of weight in the first two weeks after birth as opposed to a weight gain of 16.5 kg in calves drinking ad libitum in the first wo weeks of life [30,31]. As the calves in our study were reared in a conventional system, we did not expect any major effects on our results, although future research should consider the weight gain and include calves weighed as soon as possible after birth. It should be mentioned that the use of two different datasets in the present study needs to be considered. The farms included in these datasets did not have identical management in terms of, e.g., nutrition, heat stress abatement and genetics, while it is known that birth weight depends on both genetic and environmental factors [1]. We therefore recommend that farm-specific birth weight curves should be established.

It should also be noted that the R² of our model is moderate. Including dam parity and calf sex in the model increased the model’s explanatory power from low (R^2^ = 0.19) to moderate (R^2^ = 0.34). This indicates that only 34% of the variability in the classification of calves as small, average or large for gestational age can be explained by the combined factors of gestation length, dam parity, and calf sex. Consequently, other factors—potentially genetic, environmental, or related to management practices—likely play significant roles in determining calf size at birth. Despite the moderate R², our model retains practical utility, like for example informing management decisions or identifying high-risk calves.

It is important to note that in human medicine, the distinction is made between symmetrical and asymmetrical SGA babies, as there are differences in both etiology and prognosis. Symmetrical growth restriction is a result of a global insult early in pregnancy, while asymmetrical growth restriction with brain sparing is thought to be the result of complications later in pregnancy [14]. Asymmetrical IUGR is more often associated with utero-placental insufficiency with a redistribution of fetal blood to vital organs and a reduction in fetal cell growth rather than a reduction in cell numbers [4]. Asymmetrical IUGR babies are said to be at a higher risk for major anomalies, perinatal mortality, and overall poor neonatal outcomes (respiratory distress, sepsis,…) compared to symmetrical IUGR or AGA infants [32]. Consequently, the second aim of our study was to investigate whether the SGA calves in our study could be classified as symmetrical or asymmetrical with indications of brain sparing by gathering early postnatal body measurements as well as including the proportions of these measurements. Our results show that all body measurements are significantly smaller in SGA calves compared to their AGA and LGA counterparts. Also, LGA calves have significantly larger body measurements than the AGA calves. Next to that, we noted other similarities with human medicine when assessing the analyzed body proportions. All body proportions were larger in the SGA calves, which implies relatively longer body measurements compared to their lower birth weights and thus heart girth. Small for gestational age calves seem to be rather slender with a relatively large head compared to their AGA and LGA counterparts. LGA calves did not differ much from the AGA ones in terms of body proportions. Only the proportions including the length of their forearm and lower hindleg were significantly smaller, implying a larger body mass compared to the AGA calves. The larger head circumference relative to the heart girth (HC/HG) in SGA calves suggests head sparing. Thus, the larger HC/HG proportion suggests a somewhat later onset of the IUGR causing the birth of asymmetric small for gestational age calves. As a next step, it would be interesting to assess the morbidity and mortality results of these SGA calves as well as later performance in terms of reproduction and milk yield in comparison to normal sized calves within the same population.

The third and last goal of this study was to identify prenatal risk factors for the birth of SGA calves. To do so, we included multiple environmental and dam parameters present whilst gestating the calves in our study. As an environmental parameter, we included the THI, while dam age as well as fertility and milk yield parameters were included as maternal parameters. For nulliparous animals, age at parturition and the mean THI during the second trimester of gestation were significantly associated with the birth of SGA calves. Older (≥26 months) dams had 4.22 times higher odds to give birth to an SGA calf. These results are in line with previous research by our group reporting lower birth weights in calves born to very young (<22 months) and older (>25.5 months) nulliparous dams, suggesting the intrauterine environment to be a contributing factor to fetal growth [15]. The older dams in our study were significantly older at first insemination and needed more inseminations to become pregnant than the average and young dams. It is speculated that older heifers show suboptimal growth related to lower insulin-like growth factor 1 (IGF-1) concentrations, and that the lower IGF-1 concentrations in these older dams contribute to the lower birth weights of their respective calves [33,34,35]. A shortcoming in our study is that we did not measure IGF-1 in the dams nor in their calves. Other than dam age, a higher mean THI during the second trimester of gestation gave rise to the birth of more SGA calves, and a THI threshold of 66.8 was set, above which the odds to be born SGA was 2.75 times higher than when the second trimester THI was normal (<66.8). It has been well described that late-gestation heat stress gives rise to lower birth weight calves, by impairing placental function and reducing placental hormones [16], by altering the uterine blood flow compromising delivery of nutrients to the fetus [36], and by reducing gestation length by on average two days [37]. However, to the best of our knowledge, this is the first paper reporting a higher risk for the birth of asymmetrical SGA calves related to a higher THI during the second trimester of gestation. Research in sheep showed that mid- to late term heat stress resulted in placental insufficiency and concomitant asymmetrical IUGR [38]. The second trimester of gestation is a period of rather isometric intra-uterine growth, as it is well known that calves grow to about 40% of their birth weight during the first seven months and grow exponentially during the last two months of gestation [39]. As higher THI during this period may cause heat stress, this might reduce uterine blood flow and thus nutrient partitioning to the fetus. Since a reduction in overall nutrient availability may be detrimental for the fetus at that time during pregnancy, priority might be given to the vital organs like the brain (head sparing), leading to the birth of asymmetrical SGA calves. It would therefore be interesting to confirm our results by assessing the uterine blood flow during gestation in relation to the environmental temperature and THI. A such, reducing heat stress by cooling heifers during high THI periods might help to reduce the birth of SGA calves.

For the multiparous dams, the effect of different Milkbot^®^-parameters [23] were researched, next to fertility results of the dams, as well as the THI at different time points during gestation. Only the milk yield during gestation was significantly associated with the birth of SGA calves. The odds of birthing an SGA calf were 2.85 and 3.14 times higher in respectively low and high-producing dams in comparison to their average producing counterparts. The low-producing cows did not only have a low overall milk yield during gestation, they also had a significantly longer dry period length and showed reduced fertility, as their parturition to conception interval was significantly longer in comparison to the other milk yield groups. These animals may have suffered from (sub)clinical periparturient health issues or were genetically predisposed to lower milk yields and a lower fertility. A second limitation of our study is that we did not include sampling for metabolic or oxidative stress parameters in the dams, nor were disease events like e.g., ketosis, lameness or endometritis available. Metabolic or oxidative stress parameters in the dams’ blood could provide a link to the lower milk yield in these animals, as well as their potential disease status. The high-producing cows also had higher odds of giving birth to an SGA calf; however, they did not differ in parturition to conception interval nor in the dry period length. Our research group previously described that a calf developing in utero during lactation ‘misses’ about 446 kg of glucose and 217 kg of proteins due to the dam’s lactation, causing high-yielding dams (>7200 kg during gestation) to give birth to calves weighing on average 1 kg less than calves born to lower-producing animals [15]. Thus, we propose these high-yielding animals are less capable of partitioning nutrients between the udder and the gravid uterus, leading to higher odds of giving birth to an SGA calf. Lastly, but rather surprisingly, the very high-yielding dams (>8600 kg during gestation) did not differ from the average-producing animals. Numerically, they had the shortest parturition to conception interval as well as a shorter dry period length and are therefore capable of combining very high milk yields with good fertility and fetal growth. We suggest these very high-yielding dams are very efficient in appropriately partitioning energy and nutrients for lactation, fertility and fetal growth. They may be genetically superior to the other milk-producing groups; therefore, it would be interesting to take genomic breeding values into account to further elucidate their efficiency.

We established that the term “small for gestational” age may be applied in Holstein Friesian calves, and the results suggest that SGA calves are asymmetrical in terms of body proportions. Herd-specific birth weight curves should be made, including gestation length, calf sex and dam parity, to be able to appoint calves as SGA, AGA or LGA. Notwithstanding, the effects of being born SGA on later productive and reproductive performance remain to be described.

More research is needed on the effect of metabolic and oxidative stress in dams on the birth of small and asymmetrical calves. Also, genetics of the dams should be investigated, as there are cows that are much more efficient in combining lactation, fertility and pregnancy than others, as was apparent from this study. If such a genetic predisposition for efficiency could be found, part of the dam factors giving rise to the birth of SGA calves could be alleviated, producing average sized and healthy calves. Ultimately, while our initial results are promising, further research and field trials will be crucial in reinforcing the external validity of this method, ensuring it can be reliably applied across different breeds, environments, and over time.

## 5. Conclusions

In the present study, we defined “small for gestational age” in Holstein Friesian calves. Our findings highlight the importance of grouping calves according to gestational age as opposed to basing groups on birth weight only, as more than one third of average birth weight calves would not be classified as SGA. In accordance with human medicine, we included body measurements and proportions in our studies and could conclude that these SGA calves are asymmetrical in size with evidence of head sparing. Risk factors to be born SGA in nulliparous dams are age of the dam at parturition of ≥26 months but also when the mean THI during the second trimester is equal or higher than 66.8. In multiparous animals, both low (<5800 kg) and high (6700–8600 kg) milk yields during gestation give higher odds of birthing an SGA calf. However, very high-yielding animals do not seem to give rise to more SGA calves and are thus thought to be very efficient in terms of energy partitioning for lactation, fertility and pregnancy.

## Figures and Tables

**Table 1:** Distribution of small, average and large for gestational age (SGA, AGA, LGA) calves within calf sex and dam parity categories.

	Category	
	Calf Sex—*n* (%) *	
	Female (N = 810)	Male (N = 784)
SGA	110 (13%)	53 (7%)
AGA	662 (82%)	611 (78%)
LGA	38 (5%)	120 (15%)
	Dam Parity—*n* (%) *	
	Nulliparous (N = 540)	Multiparous (N = 1054)
SGA	105 (19%)	58 (5%)
AGA	420 (78%)	853 (81%)
LGA	15 (3%)	143 (14%)

* Percentage of SGA, AGA and LGA calves within each category.

**Table 2:** Distributions of small, average and large for gestational age (SGA, AGA, LGA) within calf sex and dam parity categories in the optimized model.

	Category	
	Calf Sex—*n* (%) *	
	Female (N = 810)	Male (N = 784)
SGA	84 (10%)	82 (10%)
AGA	639 (79%)	623 (80%)
LGA	87 (11%)	79 (10%)
	Dam Parity—*n* (%) *	
	Nulliparous (N = 540)	Multiparous (N = 1054)
SGA	55 (10%)	111(11%)
AGA	428 (79%)	834 (79%)
LGA	57 (11%)	109 (10%)

* Percentage of SGA, AGA and LGA calves within each category.

**Table 3:** Cross-table comparing number of calves in each birth weight (BW) group vs. gestational age group (N = 508).

	SGA *	AGA *	LGA *	Total
Low BW	54	18	-	72
Average BW	24	369	20	413
High BW	-	11	12	23
Total	78	398	32	508

* Gestational age groups: small, average and large for gestational age (SGA, AGA, LGA).

**Table 4:** Comparison of body measurements and body proportions between small, average and large for gestational age (SGA, AGA, LGA) calves. Proportions are defined as a percentage of heart girth (body measurement/heart girth × 100).

Measurement/Proportion	Mean ± SD			
	SGA	AGA	LGA	*p*-Value *
N	78	398	32	
Weight (kg)	35 ± 3.5 ^a^	43 ± 4.1 ^b^	49 ± 3.8 ^c^	<0.001
Heart girth (cm)	76.2 ± 3.44 ^a^	80.4 ± 3.01 ^b^	83.7 ± 3.18 ^c^	<0.001
Withers height (cm)	74.1 ± 2.91 ^a^	77.0 ± 2.82 ^b^	79.4 ± 2.48 ^c^	<0.001
Diagonal length (cm)	70.9 ± 3.41 ^a^	73.4 ± 3.20 ^b^	75.9 ± 2.85 ^c^	<0.001
Head circumference (cm)	47.0 ± 1.93 ^a^	48.9 ± 2.14 ^b^	50.4 ± 1.94 ^c^	<0.001
Length forearm (cm)	27.1 ± 1.05 ^a^	28.2 ± 0.98 ^b^	28.8 ± 0.89 ^c^	<0.001
Length lower hindleg (cm)	33.9 ± 1.31 ^a^	35.3 ± 1.23 ^b^	35.9 ± 1.16 ^c^	<0.001
WH/HG	97.3 ± 3.48 ^a^	95.9 ± 3.33 ^b^	95.0 ± 3.43 ^b^	<0.001
DL/HG	93.1 ± 3.59 ^a^	91.3 ± 3.53 ^b^	90.8 ± 3.74 ^b^	<0.001
HC/HG	61.7 ± 2.12 ^a^	60.8 ± 2.27 ^b^	60.3 ± 2.27 ^b^	0.001
FA/HG	35.6 ± 1.28 ^a^	35.1 ± 1.06 ^b^	34.5 ± 1.36 ^c^	<0.001
LHL/HG	44.5 ± 1.71 ^a^	44.0 ± 1.47 ^b^	43.0 ± 1.78 ^c^	<0.001

* Different superscripts indicate significant differences between SGA, AGA and LGA calves (*p* < 0.05) WH, withers height; HG, heart girth; DL, diagonal length; HC, head circumference; FA, length of the forearm; LHL, length of the lower hindleg.

**Table 5:** Logistic regression model of small for gestational age (SGA) vs. average for gestational (AGA) calves, with herd as random effect (N = 168).

Fixed Effect	N	% SGA	Odds Ratio	95% CI	*p*-Value
Dam age					
Young (<23 mo)	66	15.2%	1.39	0.53–4.37	0.493
Average (23–26 mo)	84	13.1%	Ref.		-
Old (≥26 mo)	18	33.3%	4.22	1.20–14.4	0.021
Trimester 2 THI			1.07		0.032

CI, confidence interval; Ref., reference; THI, temperature–humidity index.

**Table 6:** Logistic regression model of small for gestational age (SGA) vs. average for gestational age (AGA) with herd as random effect (N = 302).

Fixed Effect	N	% SGA	Odds Ratio	95% CI	*p*-Value
Milk yield during gestation					
Low (<5800 kg)	69	20.3%	2.85	1.04–8.64	0.049
Average (5800–6700 kg)	75	9.1%	Ref.		-
High (6700–8600 kg)	86	20.9%	3.14	1.20–9.37	0.027
Very high (>8600 kg)	72	13.9%	2.02	0.66–7.44	0.274

CI, confidence interval; Ref., reference.

## Data Availability

The datasets presented in this article are not readily available because the data are part of an ongoing study. Requests to access the datasets should be directed to the corresponding author.

## Supplementary Materials

The following supporting information can be downloaded at: https://www.mdpi.com/article/10.3390/ani14142125/s1, File S1: Body measurements in Holstein Friesian Calves.
