# Supplementary material for: Small for Gestational Age Calves: Part I—Concept and Definition, Contributing Prenatal Factors and Neonatal Body Morphometrics in Holstein Friesian Calves
Source: Animals (Basel). 2024 Jul 21;14(14):2125. doi: 10.3390/ani14142125 (PMC11273420; doi:10.3390/ani14142125)

**Supplementary File S1: Body measurements in Holstein Friesian Calves**

Heart Girth (HG)

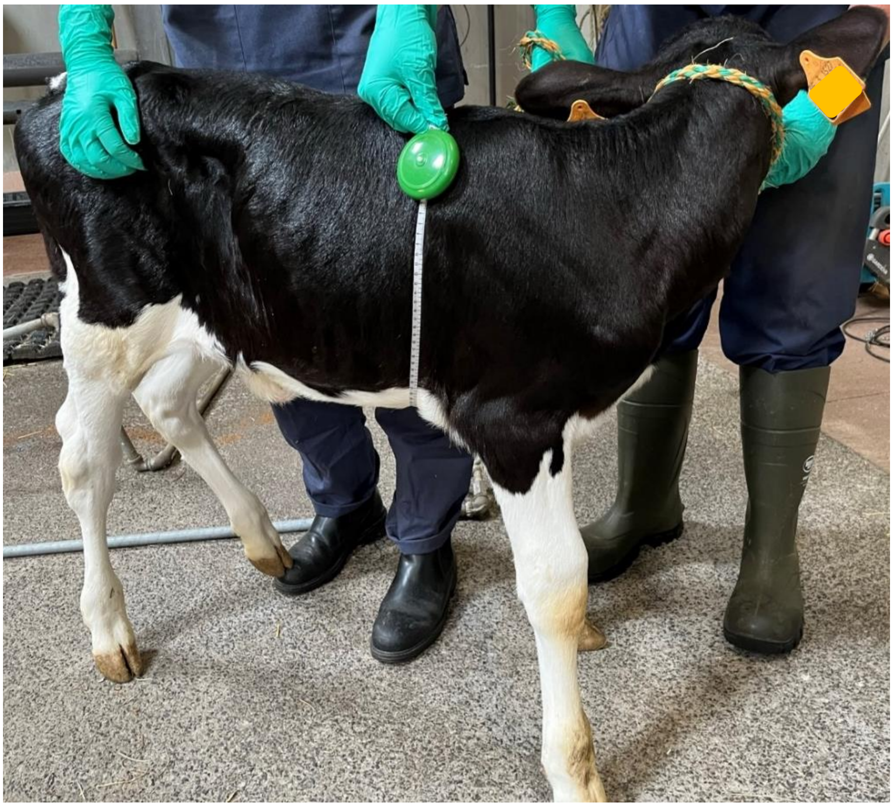

Withers Height (WH)

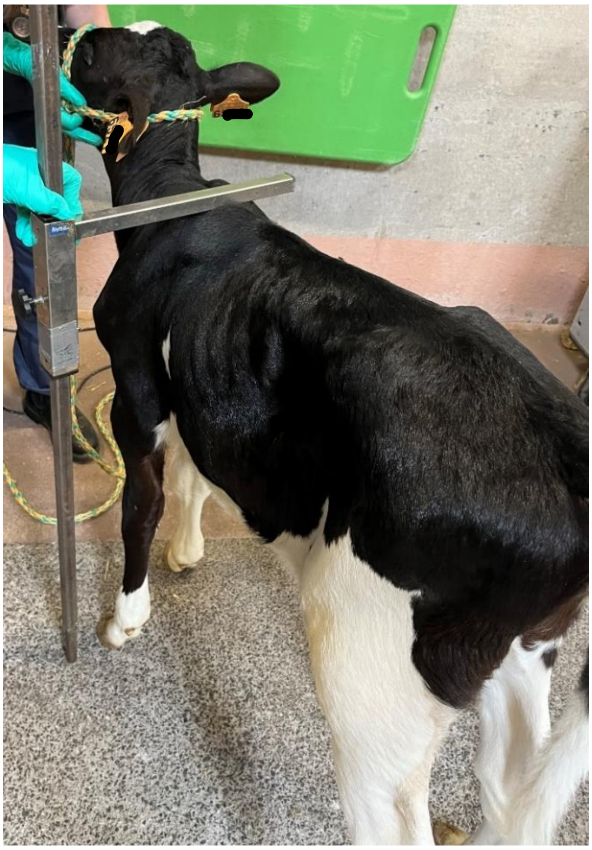

Diagonal Length (DL)

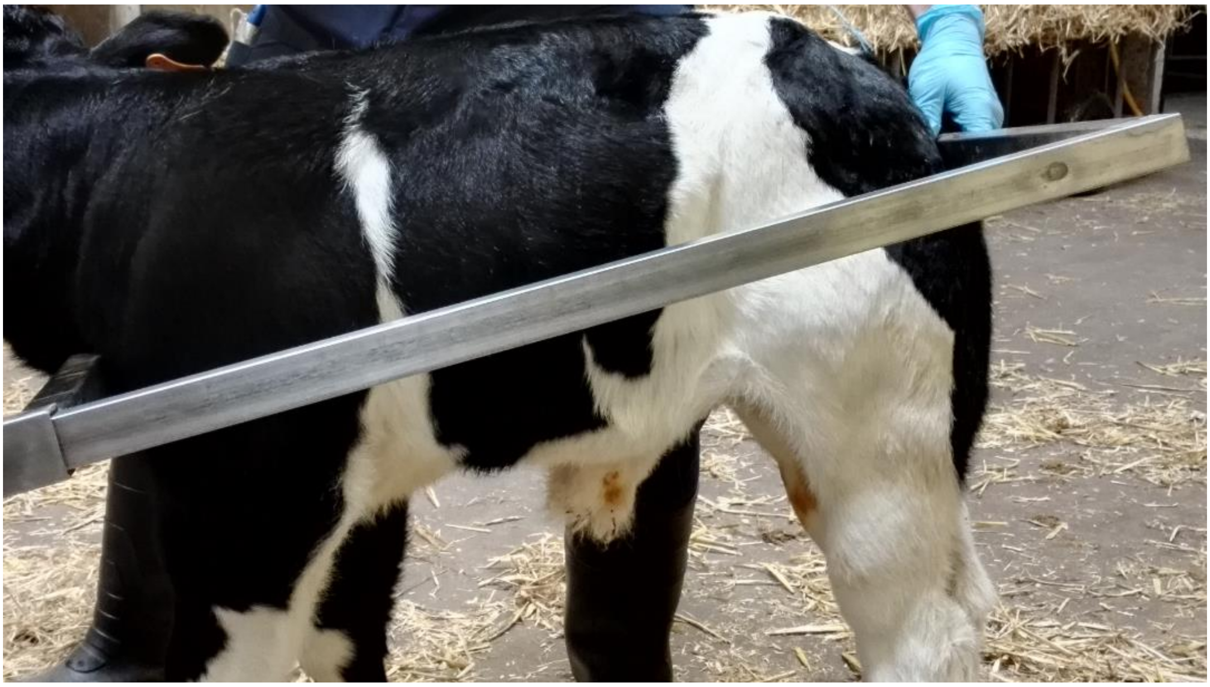

Hip Width (HW)

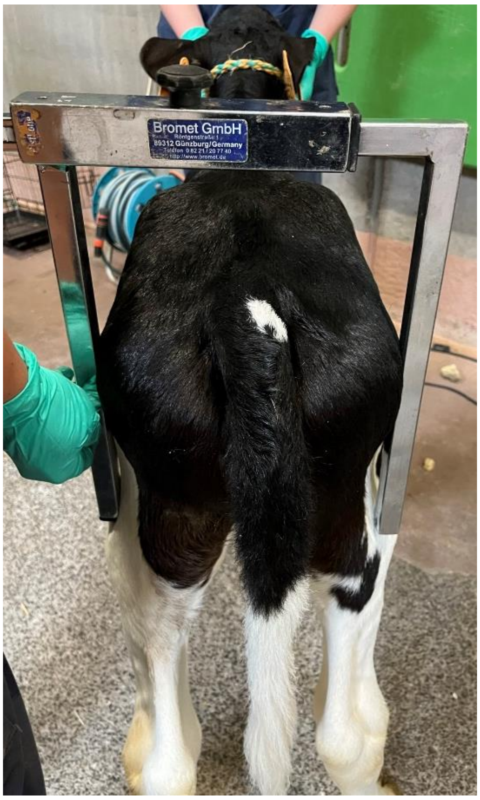

Shoulder Width (SW)

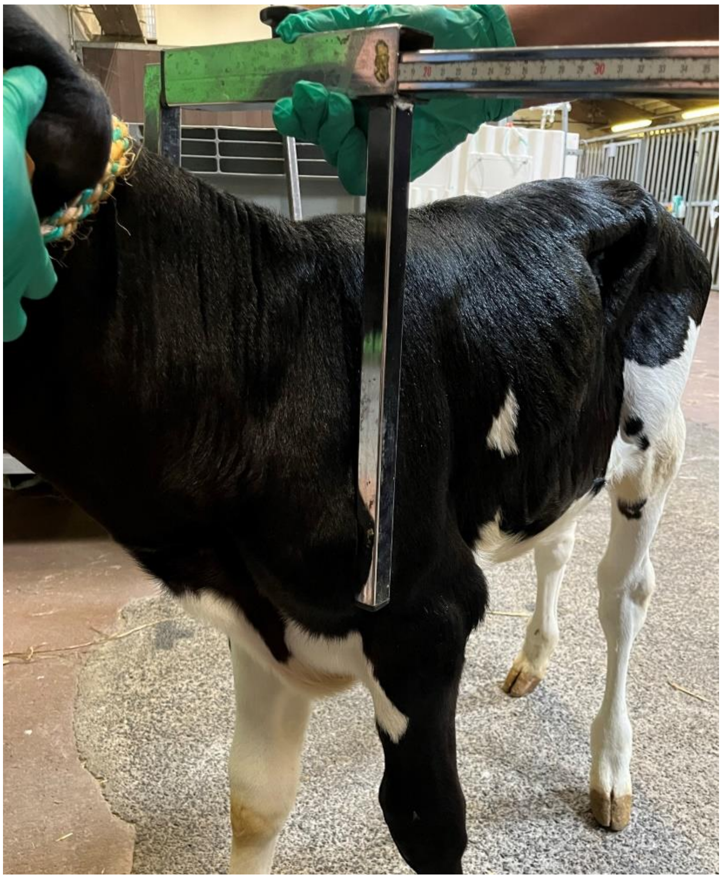

Head Circumference (HC)

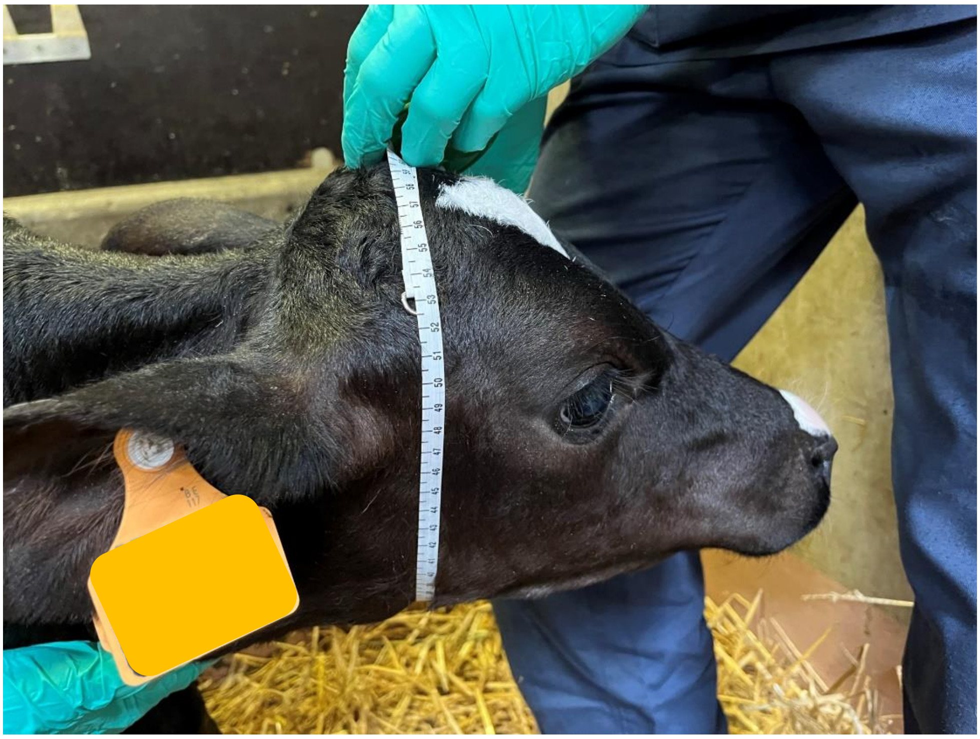

Forearm Length (FA)

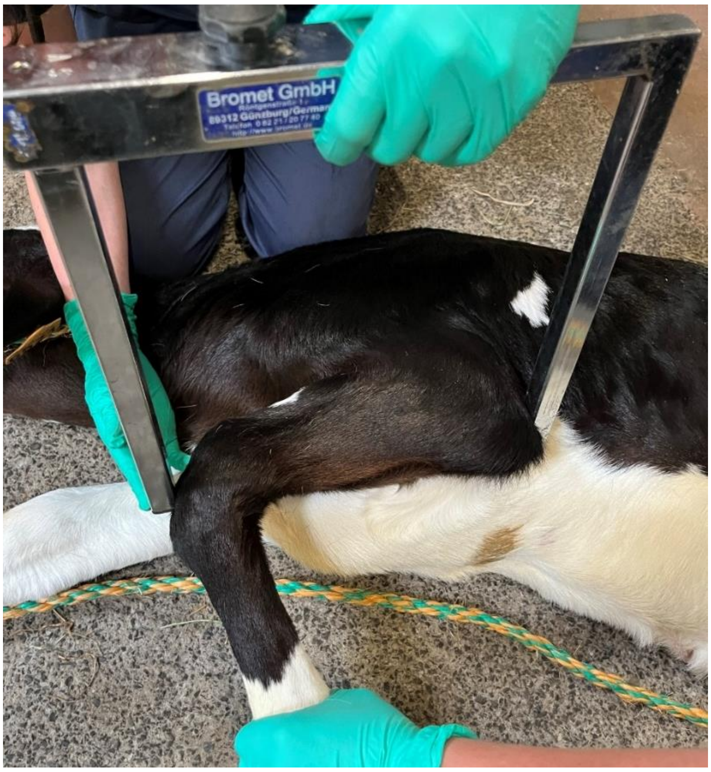


Length of the Lower Hindleg (LHL)

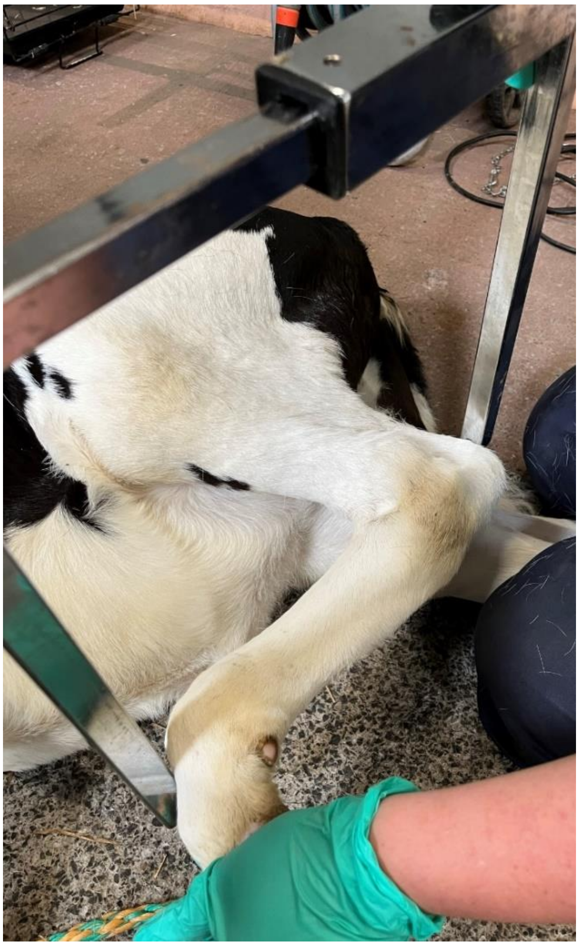

Supplement: Supplementary file 1 [file animals-14-02125-s001.zip › animals-3091261-supplementary.pdf]
